# Developing a User Reported Measure of Care Co-ordination

**DOI:** 10.5334/ijic.2469

**Published:** 2017-03-31

**Authors:** Helen Crump, Jenny King, Chris Graham, Ruth Thorlby, Veena Raleigh, Don Redding, Nick Goodwin

**Affiliations:** 1 Nuffield Trust, UK; 2 Picker Institute Europe, UK; 3 The King’s Fund, UK; 4 Natural Voices, UK; 5 International Foundation for Integrated Care, UK

**Keywords:** Integrated care, chronic condition, multimorbidity, care co-ordination, patient experience, service user experience

## Abstract

**Introduction:**

Older people with chronic conditions often receive poor care because of the fragmented way in which their services are delivered from multiple sources. Providers have limited tools to directly capture the views of older people about their experiences of care co-ordination. The study aim was to design and test a survey tool to capture the experiences of older people with chronic conditions regarding how well their health and (where applicable) social care was co-ordinated.

**Method:**

To inform the questionnaire development, we reviewed the literature on existing surveys and care co-ordination theory, and on the health status of our target audience (people aged 65 or over with one or more chronic conditions and not in hospital or residential institutions). We also consulted stakeholders including those working in health and social care services and those with expertise in the subject area. We grouped questions around experiences of care in three dimensions: care in the home environment, planned transitions in care and unplanned situations. We also designed the questions so they could be mapped onto three recognised dimensions of continuity of care – management continuity, information continuity and relational continuity – as articulated in the international literature. The questionnaire was tested using focus groups and cognitive interviews and piloted with people aged 65 and over with at least one chronic condition, using a postal survey. We used service user records in 32 general practices located in four areas and a population database held by one local authority in England as the sampling frame.

**Results:**

The pilot achieved an overall response rate of 27.6% (n = 562 responses). Ninety five percent of respondents answered 30 or more of the 46 questions and three respondents answered fewer than 10 questions. Twenty four items achieved one or more positive correlations greater than 0.5 with other survey items and four instances of positive associations greater than 0.7 were found.

**Discussion/conclusion:**

The growing focus on care co-ordination demonstrates the need for a tool that can capture the experiences of patients accessing care across organisational and professional boundaries, to inform the improvement of care co-ordination activities from a patient perspective. Early results suggest that our tool may have a contribution to make in these areas. However, more work is required to test the efficacy of the tool on a larger scale and in different settings, and to find ways of improving response rates.

## Introduction, comprising background and problem statement

Patients and service users with multiple chronic conditions often receive poor care because they access services from multiple different providers, and these services are often badly coordinated. As populations age and the prevalence of chronic ill health rises worldwide, it is becoming crucial to improve the coordination of care both within and between health care organisations, and between providers of health and social care [1].

It is now recognized internationally that effective coordination is a hallmark of high quality care. In Europe, countries such as Germany and the Netherlands have been working to improve coordination of services across providers since the 1990s, while in Austria, efforts to integrate services have been underway since the 2000s [1]. In the US, integration is essential to the delivery of the Triple Aim: improved health outcomes, better patient experience and more cost effective care [2]. There is much global interest in the progress of the Accountable Care Organisations (ACO) in the US, as well as special needs plans for patients with complex care needs being pioneered by Centers for Medicare and Medicaid Services [3].

In England, the government has accepted the need for better coordination and ‘integration’ of publicly funded health and social care services, and the 2012 Health and Social Care Act has created new legal duties for both planning and purchasing bodies (known Clinical Commissioning Groups, or CCGs), and providers and regulators to take specific action to secure integrated services where this is beneficial to patients. There has been a specific call for ‘a new generation of patient reported experience measures that evaluate patients’ experiences across whole journeys of care, and within and between services’ [4]. Within England, the situation is further complicated, as health care services and social care services are administered separately at a local level.

Understanding user experience is an important way for providers to assess how well the full package of care is meeting users’ needs. But measuring user experience of receiving integrated care from multiple sources can be hampered because of the limited availability of tools for capturing user and carer experience of care co-ordination across organisational boundaries. In contrast with existing tools designed to understand patient experience of recent episodes of care, such as the inpatient survey [5], now standard practice in England, there is a dearth of tools for measuring community-based patients’ experience of care accessed from multiple providers.

The need for such tools is pressing: multimorbidity is a growing challenge for health systems and there is consensus that improvement is needed to ensure people accessing multiple services receive care that is truly joined up eg. [6,7].

For this growing cohort of multi-morbid patients there are both clinical and structural reasons why many existing tools are generally not well suited for capturing their experiences, which can span multiple providers and pathways. Preparatory research by the project team [8] found that although various user-reported and service-reported measures of quality and performance were in use within the English NHS (for example, the Personal Social Services Adult Social Care Survey, the NHS Inpatients Survey and Hospital Episode Statistics), “no single data source or measure [was] currently suitable for measuring people’s experiences of integrated care comprehensively across and within health and social care settings” [8, p. 7].

Although some existing NHS health and social care surveys contain some questions on integrated care, there were gaps, particularly in relation to the experiences of people as they move between services. A four-point patient reported measure of integration in health care delivery which has recently undergone cognitive testing [9] uses four questions to explore patient views about information sharing, consistency of advice, mutual respect and role clarity. The authors describe the tool as having been designed to be completed within a minute, but this means it is unlikely to be able to provide a detailed picture of what is happening at different points within a patient’s journey.

There are methodological challenges in understanding user experience of care co-ordination. Haggerty et al’s 2013 analysis of 33 qualitative studies of patients’ experience of care [10] describes three dimensions of care continuity that affect patients’ experience: relational, informational and management continuity. The authors find that “for patients, continuity of care is expressed as security and confidence rather than seamlessness”, that “coordination and information transfer between professionals are assumed unless proven otherwise” and that “care plans help clinician coordination but are rarely discerned as such by patients”. Instead, for patients “knowing what to expect and having contingency plans provides security” [10, p. 262].

The authors found that patients tend to assume that communication has taken place between professionals involved in their care. However, patients can accurately report on failures and gaps [10, p. 269]. The authors suggest a range of practical approaches to measuring continuity, including whether patients feel their role and ideas are acknowledged by care providers; whether they have experienced care management problems that have resulted in discontinuities; or lack of role clarity; whether they have received conflicting advice or information; whether they feel they have been provided with adequate information; and whether there is a relationship with an individual clinician who has developed a comprehensive knowledge of the patient as a whole person and uses that in managing their condition [10, p. 266–267].

Aller et al [11] surveyed 1500 patients to gauge their experience of relational, informational and managerial continuity of care. They found that about a quarter of patients surveyed did not “perceive” appropriate transfer of information. And although most perceived that care was consistent among providers, reasons for perceiving that this was not the case related to issues such as “transfer of information and consistency of instructions among professionals” [11, p. 296]. This further reinforces the idea that patients are better able to identify tangible gaps in continuity.

A final consideration when preparing a survey tool is the appetite for such a tool among professionals and service users, and the degree to which uptake rates could be affected by survey fatigue, as identified by Coulter et al [12].

In summary, the literature shows that organising services effectively for patients with more than one chronic condition is an area of growing importance. The effectiveness of these approaches to care co-ordination can be measured across several different dimensions. Survey questions must be framed to elicit an accurate picture of what service users have actually experienced, rather than eliciting assumptions they might make about the quality of interactions between professionals that they have not observed. Surveys must also be designed with an awareness of the trend of falling response rates, as identified by Coulter et al [12].

## Theory and methods

Ethical approval for this research was obtained from the English NHS Health Research Authority and the project was funded by the Aetna Foundation.

The primary purpose of the study was to design and test a survey tool that would capture perceptions of care coordination in older service users with one or more chronic conditions. Our ultimate aim was that it would support quality improvement in the way care is co-ordinated across services for users with one or more chronic conditions; enable care providers and purchasers to assess where care is fragmented; evaluate the success of interventions designed to improve coordination and provide feedback on aspects of care coordination to older people that could be improved.

### Preparatory stakeholder consultation

The initial design of the tool was informed by three consultative discussion groups with national stakeholders including charities, central government and non-governmental organisations, regulators, professional bodies, think tanks and academic institutions in England [8]. During these meetings, participants described barriers to achieving and measuring integrated care including:

- The absence of timely and appropriate information sharing within, between and across services and sectors
- The lack of a shared language between health and social care
- The misalignment of incentives for providers in different services and sectors
- Tensions, particularly described by discussion group participants in social care but also present in health care, between the multiple standards services must adhere to and the challenges of delivering high quality co-ordinated care

All of the stakeholders consulted felt any measure or indicator developed must be able to capture experiences of where care crosses boundaries between health and social care, or within each sector. They felt some transitions – such as from child to adult services, from health to social care and from curative to palliative to end-of-life care - were particularly important. A majority of stakeholders agreed that integrated care indicators should ideally be aimed at the general population, but the following subgroups (and their carers) were identified during the consultation as promising areas to focus on initially:

- People with dementia
- People with long-term conditions, physical and mental
- People who have learning disabilities
- People who are at the end of life
- Older people
- Children

The stakeholder consultation revealed that participants understood that any indicators developed from a survey tool may not be able to provide a high level of detail. However, they were concerned that the tool should be useful at a local level. They identified specific ways to achieve this, such as that the tool should:

- Be capable of driving improvement
- Be useful and “actionable” for commissioners
- Encourage health and social care professionals to start communicating and working together better
- Benefit the wider health and social care system, for example by creating an enhanced working environment and/or driving financial and efficiency savings

### Tool design

Taking into account this feedback, and the results of the literature review, we decided to frame the tool as a user-reported postal survey targeting older people with at least one chronic condition, in keeping with advice from the stakeholder consultation that the survey should ideally be suitable for use in a general population context.

We chose to open the questionnaire with outcome-based questions focused on the respondent’s quality of life, rather than questions about service provision. This decision was informed by the results of “I’m Still Me”, research by National Voices, Age UK and UCL Partners, based on focus groups and interviews with older people older people living with frailty [13], itself based on National Voices’ earlier Narrative for Person-centred Co-ordinated Care [14]. This found that this group prioritised living the life they wanted to the best of their ability; setting and achieving goals relating to care; deciding on their support and how to receive it; having carers’ needs taken into account and having control over their planning and support. Patients in this age group were found to have predominantly non-service related goals (Table 1).

**Table 1:** I statement [goals] of older patients in National Voices study ‘I’m Still Me’ [13].

Theme	I statement

Independence	I am supported to be independent
	I can do activities that are important to me
	I am recognised for what I can do rather than making assumptions about what I cannot
	My family are recognised as being key to my independence and quality of life
Community Interactions	I can maintain social contact as much as I want
Decision making	I can make my own decisions, with advice and support from family, friends or professionals if I want it
Care and Support	I can plan my care with people who work together to understand me and my carer(s), who allow me control, and bring together services to achieve the outcomes important to me
	Taken together, my care and support help me live the life I want to the best of my ability
	I can build relationships with people who support me

The remainder of the survey tool was structured by drawing on international research into concepts of care co-ordination. Where relevant, we based other survey questions on items and concepts already in use internationally. Existing surveys used in England and internationally that we consulted included the Adult Social Care Survey [15], the NHS Inpatient Survey [5], the Cancer Patient Experience Survey [16], the GP Patient Survey [17] and the Care Transitions Measure 15 [18].

We chose to group questions around three core areas where care co-ordination is considered important to improving quality of care and outcomes, asking about experiences at three different points of a person’s life with chronic illness:

– Care in the home environment
– Planned transitions in care
– Unplanned situations/emergency admissions to hospital

The selection of these three core areas for care co-ordination was based on findings from several studies that highlighted their importance in improving quality of care to older people (e.g. [19,11]). We grouped questions so that they also loosely mapped onto three widely understood dimensions of continuity of care – management continuity, information continuity and relational continuity [10]. See Tables 2 and 3.

**Table 2:** Survey question themes and how they map onto the different dimensions of care continuity identified in the research.

	Care in the home environment	Planned transitions in care	Unplanned situations/emergency admissions

Relational continuity	“knows who to contact with questions bout condition(s)”	“home situation considered when planning discharge”	“patient can identify first person they would contact if they needed help”
	“has single named professional”		
Informational continuity	“staff have provided information about available services”	“GP was informed about outcome of planned hospital treatment”	“emergency staff could quickly access information about conditions”
		“patient had enough information to be able to take care of him/herself after leaving hospital”	
Management continuity	“needs assessment carried out”	“post-discharge care plan in place”	“care received inspires confidence needs will be met in an emergency”
	“care plan in place”	“discharge care plan made patient confident they could manage their own care on leaving hospital”	
		“received expected support to manage health day to day on leaving hospital”	
I statements	“supported to make own decisions”	“patient views are taken into account when producing care plan”	N/A
Other	“Support from social services is sufficient”		

**Table 3:** Questionnaire concepts and example questions.

Questionnaire concept	Example questions

Your health and wellbeing	“On the whole, are you able to do the activities that are important to you?”
	“To what extent do you agree or disagree with the following statement: ‘I am supported by health and care staff to be as independent as I can be’”
Managing your health day to day	“Do you have a single named health or care professional who coordinates all your care and support?”
	“Do you feel that health and care staff listen to what you have to say?”
Support from social services	“At the present time, do care workers visit you as often as you need?”
Planned care	“Do you have a written care plan?”
	“Are your views taken into account when deciding what is in your care plan?”
Urgent access to health care	“Does the care you receive make you feel confident that your needs will be met if you need to access healthcare urgently?”
	“If you are feeling unwell and need help, who is the first person you contact for help?”
Hospital care	“Did hospital staff take your family or home situation into account when planning your discharge?”
	“Before you left hospital, did the hospital staff spend enough time explaining the care and support you would receive when you got home?”

### Focus groups and cognitive testing

We conducted two focus groups of eight to 10 people in our target audience, aged 65 and over with one or more chronic condition to test the survey tool. We also convened a panel of clinicians and managers from NHS provider and purchaser organisations, to establish whether they viewed the survey tool as a resource that would potentially support them in improving the quality of the services they provided or purchased.

Following the focus groups and survey validation, we undertook 29 cognitive interviews with people in our target audience. During this phase of the research, the questionnaire performed well and seemed to resonate with patients’ and service users’ experiences. Issues raised were typically around interviewees’ comprehension, interpretation, evaluation and response to questions. As a result of the cognitive testing, we removed one question, made changes (rewriting a significant part of the text) to five questions and minor changes (slight changes to wording or extra answer options) to seven questions. The final draft survey tool comprised 46 questions.

### Survey pilot

When we began piloting it was clear that significant appetite existed among purchasers and providers in England for a survey tool of this kind as we received an enthusiastic response from multiple locations.

We began pilot site recruitment by advertising the pilot to purchasers (NHS clinical commissioning groups) and making direct contact with purchasers where we knew they had an existing interest in care co-ordination. Organisations with responsibility for purchasing health or care services (referred to as “commissioning” in England) in nine locations expressed an interest in taking part in the pilot. Of the nine sites that originally volunteered to participate in the pilot, five finally went through to pilot stage.

Although pilot site recruitment and results analysis took place at a purchaser level, the survey administration took place at primary care practice level. Individual practices hold patient details, which would be needed to identify people in the target audience, and patient confidentiality requirements can prohibit patient contact details being shared outside the NHS, or between GP practices and commissioners. Surveys were sent by GP surgeries to a sample of patients in the target audience, with a reminder letter issued to non-responders after 3 weeks and a duplicate questionnaire issued to non-responders after 6 weeks.

### Analysis

The survey data were analysed and summary reports supplied to each pilot site. Through further analysis of the aggregated responses from the pilot sites, we explored unit non response (non-completion of the survey), item non-response (not completing individual questions), use of “don’t know” and “not applicable” responses, floor and ceiling effects, item correlations and free text comments.

Factor analysis was not conducted due to the low volume of data and the number of questions that would need to be removed prior to running the analysis (including those answered by less than 50% respondents.

## Results

### Survey data analysis

The pilot achieved an overall unit response rate of 27.6% (562 out of 2033 contacted). The lowest pilot site response rate was 14% and the highest pilot site response rate was 45%. Five hundred and thirty two out of 562 respondents (95%) answered 30 or more of the 46 questions in the survey. Only three respondents answered fewer than 10 questions.

The demographic profile of the respondents is set out in Table 4 below.

**Table 4:** Demographic profile of survey respondents.

Gender	

Male	54%
Female	46%
**Age range**	

65–74	41%
75–84	43%
85–94	13%
95+	1%
No reply	2%
**In general, how would you describe your health?**	

Excellent	2%
Very good	11%
Good	26%
Fair	41%
Poor	20%

When assessing how well a survey tool is working, looking for correlations between the responses to different questions can reveal the strength of relationships between different survey questions. Item correlations allow exploration of the strength of the relationship between questions. A correlation can be positive (a high score on one question results in a high score on another question) or negative (a high score on one question results in a low score on another question). The closer the value is to –1 or +1, the stronger the association is between the variables. According to Cohen [20], a correlation of magnitude 0.3 is ‘moderate’, and 0.5 is a ‘large’ effect.

In our survey, twenty four items achieved one or more positive correlations greater than 0.5 with other items in the survey. Four instances of positive associations over 0.7 were found. One question, exploring the extent to which health and care staff “bring together services that help me to achieve the outcomes important to me,” had 14 positive associations stronger that 0.5 with other questions in the survey. The question pair with the highest positive association (0.708) was “Is the care and support you receive always kept up to date with your needs?” and “Does the care and support you receive make you feel secure and confident about living with your condition?”.

The average item non response (where a respondent skips a question that they would be expected to answer) for the survey was 3.6%. Two questions in the survey had 0% item non-response, meaning no respondents skipped the question. The largest item non-response was 8.7%. Only 3% of total freetext comments made by people filling in the questionnaire (14 out of 462) related to issues with the survey.

### Identifying problematic survey questions

After analysing the survey responses, the project team identified five survey questions where further changes were required. For two of these questions, we added a “not applicable” answer option. However, for five questions, more significant attention was required. These questions are explored in detail below.

The first problematic question was “In the past year, do you remember a time when health and social care staff failed to share important information about your medical history or care (such as medicines, test results or notes) with each other?”. When answering this question, 16.7% of respondents (the highest proportion) answered “don’t know/not sure”, and 3.7% skipped the question.

During earlier cognitive testing of this question, all participants said they had found the question easy to understand, but one respondent was later found to have misunderstood, thinking that the question was about whether information had been shared with her.

There was a difference between respondents who could recall a specific event where information had not been shared and those who could not. Those in the latter category confirmed the premise put forward by Haggerty et al [10] that patients are better able to identify instances of poorly joined up care than instances of well joined up care. For example, one respondent in cognitive testing said: “This has never happened to me. I am not sure I would know if it did. You don’t know what they don’t share with you until something has gone wrong.” One further respondent thought that staff must be sharing information because they were helpful and able to look things up on a computer. It is possible that the high number of “don’t know” responses is because respondents are not confident that this has not happened, but do not have a clear enough memory of a specific incident to warrant answering yes. We concluded that this question would require re-wording or removal from any further survey.

The second question to be queried was: “To what extent do you feel that health and care professionals work well together to provide the best possible care and support?”. In this case, 16.5% of respondents answered “don’t know/not sure” and 18.3% said that the question was not applicable to them. This question had also initially been framed in the negative and had been changed during cognitive testing, after some respondents suggested that the negative component might confuse people or “trip them up”. When the question was reframed in the positive during the cognitive interviewing, all respondents who answered in the negative were able to provide an example, for instance: “It took three months to get a podiatry appointment due to lack of communication”. We concluded that this question would also require re-wording or removal.

The third question posing significant issues was: “To what extent do you agree or disagree with the following statement… ‘when care and support is planned, it happens’?” At 5.5%, this question has the eighth highest level of missing data in the survey. It also had the highest proportion of people selecting the “not applicable” option (39%), which is of concern, given that it refers to care in general and should therefore be applicable to all. Again, this question would require re-wording or removal.

Two further questions answered only by those indicating that they had received social care support also posed a challenge. These were “at the present time do care workers visit you as often as you need?” and “at the present time, when care workers visit you, do they spend the right amount of time with you?”. For both questions, a high proportion of the respondents who had indicated that they were receiving social care support (26.7% and 28.9% respectively) said that the questions were not applicable to them. This suggests either that many patients in receipt of social care were not in regular contact with care workers, or that the term “care worker” was problematic. This requires further exploration, with potential approaches being to remove the question or use a different term in place of “care worker”. Other explanations could be that respondents had received home adaptations and equipment, or day/respite care rather than regular visits, or that they had misunderstood the term “social care”.

### Problems with terminology

One further issue that emerged in general feedback on the survey was that respondents interpreted questions using the form of words “health and social care professionals” as meaning interactions specifically between health care staff and social care staff, rather than between different types of health care staff and/or social care staff. This is likely to be a particular problem in the UK context where health and social care are seen as separate services.

## Discussion

The reason for undertaking this project was that no single tool existed in England that adequately captured user experience of care co-ordination, and that capturing such information could provide valuable insights for providers and purchasers aiming to improve their services.

We developed a tool that aimed to capture the perceptions of older people with a least one chronic condition as they experienced care from different providers. The tool performed well during piloting. In particular, we were satisfied with the results of the correlation analysis, and with the low item non response rate and the high proportion of people who answered 30 or more questions.

However, we experienced difficulty in recruiting pilot sites, and a low survey response rate. We were not able within the scope of this research to follow up non-responders to establish why they had not completed the survey.

### Challenges relating to the pilot

The largest practical challenge was the difficulty in recruiting pilot sites. Recruitment was already difficult because of the volume of new initiatives, the existing workloads that purchasers (CCGs) and general practices were facing and expectations to generate efficiencies throughout the NHS at the time of the pilot. However, we suspect recruitment was made harder because the point in the system where benefit might be gained from the survey results was different from the point where the burden of additional workload would fall. In order to ensure that piloting went ahead, we decided to offer participating pilot locations a £200 incentive payment to compensate for the time of GP practice staff who were tasked with identifying patients in the target group and mailing out surveys.

It would be beneficial to test this hypothesis by piloting the tool in more locations where this distinction does not exist. Indeed, this seems to have been less of an issue at our local authority pilot site, where the patient list was held by the organisation that would make use of the final outputs, and in pilot locations where there was strong buy-in from family doctors and local administrative teams into the benefits of integrated care.

Pilot site recruitment began at a time when the English NHS was under significant financial pressure, (which persists), and when several high profile national pilot programmes were already underway. Additionally, the only route to sampling patients in most cases was via the GP practice list. Family doctors in England are not directly managed by local purchasing organisations such as clinical commissioning groups and local councils. Instead, the majority operate as small independent businesses with their contractual agreements held centrally with the national NHS via the national arm’s length body NHS England. This means practices can have insufficient administrative support to enable them to participate in multiple pilot schemes, and purchasers wishing to collect patient experience data at a population level have limited abilities to enlist the support of practices, if they are unwilling.

These factors meant fewer expressions of interest translated into live pilots than initially hoped, and in some locations where pilots did take place, there were delays in distributing questionnaire packs. The exception to this rule was our one local government pilot site, where the local authority had built its own patient and service user database and was able to administer the pilot in-house. This is potentially significant in an international context where, if the objective of the body using the survey is to survey large numbers of patients in a broad target group, it might be even harder to identify them via family doctor lists.

### Low response rate

The low response rate for the survey was also disappointing. Potential explanations for this include the general survey fatigue identified by Coulter et al (2014), the difficulties we experienced in running the pilot (explored above) or the length of the questionnaire. Although this was not a short survey, we felt it was comparable with other surveys in use in England and internationally that assess patients’ experiences (eg. [21]), and it is shorter than the NHS Inpatient Survey [5], which in 2014 had 78 questions, but nevertheless achieved a response rate of 47% [22].

### Target audience

It is possible that the low response rate was related to our target audience. We had opted to pilot the tool among people aged 65 and over with one or more chronic condition in order to ensure that a broad group received the survey. However, relatively few of the respondents (13% and 1%) were in the 85-94 and 95+ age ranges respectively, and nearly four in 10 respondents (39%) rated their health as either good, very good or excellent. Therefore, the survey questions, which assume a certain level of need and intensity of service use, may not have been appropriate to a proportion of our survey respondents, for example those with only one or two chronic but stable conditions, which require infrequent contacts with service providers.

Responses to free text questions appear to support the notion that some of the survey recipients felt that their health was sufficiently good and that the survey did not apply to them, for instance: “*I have left a lot of questions blank as they do not apply to me. I do not require any care”; “Found this survey awkward to fill in – as it seems to refer to someone with a major problem, not like myself. I live a normal life with no restrictions.”* For subsequent use of the tool, we would propose that a narrower, more complex, multi-morbid target audience was selected, perhaps determined by the quality improvement question that the organisation deploying the tool was seeking to answer. Service users surveyed would need to have a minimum of two chronic conditions and to require some support to co-ordinate their care.

### Semantic challenges

When designing and piloting the survey tool, we repeatedly encountered challenges stemming from the fact that terminology related to integrated care is used differently in different countries. In particular, we found the terms “integrated care”, “care co-ordination”, “continuity of care” and “care transitions” meant different things to different people, depending on the contexts in which they were operating.

In order to address this challenge, we developed our own definitions for these terms, which we used throughout the project. For instance, we used “integrated care” as an umbrella term for different integration strategies, and “care co-ordination” as an active process by which services are delivered by multiple providers with and to users. This enabled us to think more clearly about which specific issues we were seeking to explore in designing the survey tool and helped us to make better use of existing frameworks and typologies for describing care continuity. However, we are aware that different definitions are in use outside the project, and we will need to take this into consideration when describing our work to potential users, and particularly when discussing it in an international context.

### Opportunities to translate survey findings into quality improvement strategies

Although testing the extent to which the survey tool was able to drive quality improvement in piloting organisations was beyond the scope of this research, this is an area we would like to explore in future testing of the survey tool. One immediate challenge in this regard will be the fact that findings from the survey tool apply at a cross-organisational level, rather than relating back to the specific decisions of individual provider organisations. This is likely to mean that the tool will find greatest use as a diagnostic device, enabling those involved in providing care to understand the impact of their collective decisions from a service user perspective. The survey tool would therefore be best supported by an additional process of reflection that enables providers to come together and consider what it is about their pathways that makes service users respond in particular way. This also reinforces the point that the survey tool will not have a role as a performance management device.

### Limitations of the research

The piloting tested the application of the survey as a postal tool. The tool was tested with one target audience of people aged 65 and over with one or more chronic conditions. The tool was tested as a one-off assessment of patients’ experience of care coordination, therefore we have not yet been able to establish what the minimum time would be between survey completions in order to demonstrate change. This is something we would hope to pursue in future work. The piloting focused on the performance of the tool itself, rather than the ability of providers and commissioners to use findings from the survey to improve the quality of services. Therefore, we have not been able to assess the performance of the tool in the following scenarios:

– In settings other than completion by post (i.e. face-to-face, telephone)
– In settings other than user's residences
– In target audiences other than people aged 65 and over with one or more chronic conditions
– As a tool to measure improvements in experience achieved via specific interventions (i.e. by surveying patients and service users at the start and end of the intervention)
– As a tool for enabling providers and commissioners to change how services are provided in order to improve their quality (i.e. the applicability of the tool’s findings)

We recognise there are further opportunities for development, specifically exploring the tool’s performance in other forms than a postal survey, or with people of varying cognitive abilities, or with those for whom English is a second language. In addition, those in residential care have not been surveyed, so we do not know how the tool will perform in these contexts. We have also not tested the tool with the carers of patients in relevant target audiences. It is likely that further testing and development will be necessary in order to adapt the tool for use with carers, younger age groups and those for whom English is not the first language.

## Conclusion

The continued focus on care co-ordination, both within England and internationally, demonstrates the need for a tool that can capture the experiences of patients accessing care across organisational and professional boundaries. This tool has the potential to serve this purpose in support of improving care co-ordination. Our survey instrument’s performance in piloting to date suggests that when tested further, it will be able to offer some insights to purchasers and providers about how patients experience their efforts to coordinate services across boundaries of provision. The survey should also provide some information about how well services are supporting patients and service users in achieving their own life goals. In this respect, the survey tool has the potential to occupy a role not currently filled within an English context.

However, further work will be required to test the tool with different target audiences and in different contexts (eg. face-to-face, by telephone), to assess the extent of its potential application. It will also be important in further research to assess the extent to which survey data provides information that providers and purchasers can use to make specific changes to improve the quality of their services.

## References

[1] Nolte E, Knai C, Hofmarcher M (2012). Overcoming fragmentation in health care: chronic care in Austria, Germany and the Netherlands. Health Ecomomics, Policy and Law.

[2] Institute for Healthcare Improvement (2009). Triple Aim: concept design. http://www.ihi.org/Engage/Initiatives/TripleAim/Documents/ConceptDesign.pdf.

[3] Martyn H, Davis K (2014). Care coordination for people with complex care needs in the US: a policy analysis. International Journal of Care Coordination.

[4] NHS Future Forum (2012). Integration: a report from the NHS Future Forum. https://www.gov.uk/government/uploads/system/uploads/attachment_data/file/216425/dh_132023.pdf.

[5] NHS Inpatient Survey (2014). NHS Surveys. http://www.nhssurveys.org/Filestore//Inpatients_2014/IP14_Questionnaire_v2.pdf.

[6] Barnett K, Mercer S, Norbury M, Watt G, Wyke S, Guthrie B (2012). Epidemiology of multimorbidity and implications for healthcare, research, and medical education: a cross-sectional study. Lancet.

[7] Roland M, Paddison C (2013). Better management of patients with multimorbidity. British Medical Journal.

[8] Graham C, Killpack C, Raleigh V (2013). Options appraisal on the measurement of people’s experiences of integrated care, Picker Institute Europe. http://www.pickereurope.org/wp-content/uploads/2014/10/Options-appraisal-on...-integrated-care.pdf.

[9] Elwin G, Thompson R, Roshen J (2015). Developing IntegRATE: a fast and frugal patient-reported measure of integration in health care delivery. International Journal of Integrated Care.

[10] Haggerty J L, Roberge D, Freeman G K (2013). Experienced continuity of care when patients see multiple clinicians: a qualitative metasummary. Annals of Family Medicine.

[11] Aller M B, Vargas I, Waibel S, Coderch J, Sanches-Perez I (2013). A comprehensive analysis of patients’ perceptions of continuity of care and their associated factors. International Journal for Quality in Health Care.

[12] Coulter A, Locock L, Ziebland S, Calabrese J (2014). Collecting data on patient experience is not enough: they must be used to improve care. British Medical Journal.

[13] National Voices, Age UK and UCL Partners (2014). I’m still me. http://www.nationalvoices.org.uk/publications/our-publications/im-still-me.

[14] National Voices (2013). A Narrative for Person-centred Co-ordinated Care. http://www.nationalvoices.org.uk/publications/our-publications/narrative-person-centred-coordinated-care.

[15] Health and Social Care Information Centre (2015). Adult social care survey. http://www.hscic.gov.uk/ascs1516.

[16] Quality Health (2014). National Cancer Patient Experience Survey. https://www.quality-health.co.uk/resources/surveys/national-cancer-experience-survey/2014-national-cancer-patient-experience-survey.

[17] GP patient survey (2017). GP Patient survey. https://gp-patient.co.uk/.

[18] Care Transitions (2017). Care transitions measure 15. http://caretransitions.org/tools-and-resources/.

[19] Goodwin N (2014). Providing integrated care for older people with complex needs, The King’s Fund. https://www.kingsfund.org.uk/sites/files/kf/field/field_publication_file/providing-integrated-care-for-older-people-with-complex-needs-kingsfund-jan14.pdf.

[20] Cohen J (1988). Statistical power analysis for the behavioral sciences.

[21] Safran D G, Kosinski M, Tarlov A (1998). The Primary Care Assessment Survey: tests of data quality and measurement performance. Medical Care.

[22] Care Quality Commission (2015). Trends in the adult inpatient survey 2005–2014. Care Quality Commission.

